# Early-onset cardiac dysfunction following allogeneic haematopoietic stem cell transplantation

**DOI:** 10.1136/openhrt-2022-002007

**Published:** 2022-05-19

**Authors:** Shohei Moriyama, Mitsuhiro Fukata, Michinari Hieda, Taku Yokoyama, Goichi Yoshimoto, Hitoshi Kusaba, Yasuhiro Nakashima, Toshihiro Miyamoto, Toru Maruyama, Koichi Akashi

**Affiliations:** 1 Department of Haematology, Oncology and Cardiovascular Medicine, Kyushu University Hospital, Fukuoka, Japan; 2 Department of Medicine and Bioregulatory Science, Graduate School of Medical Sciences, Kyushu University, Fukuoka, Japan; 3 Department of Hematology, Faculty of Medicine, Institute of Medical Pharmaceutical and Health Sciences, Kanazawa University, Kanazawa, Ishikawa, Japan; 4 Center for Health Sciences and Counseling, Kyushu University, Fukuoka, Japan

**Keywords:** cardiomyopathies, heart failure, systolic, risk factors

## Abstract

**Objective:**

Heart failure following allogeneic haematopoietic stem cell transplantation (allo-HSCT) is a serious complication that requires early detection; however, the clinical implications of early-onset cancer therapy-related cardiac dysfunction (CTRCD) following allo-HSCT remain unclear. We investigated the determinants and prognostic impact of early-onset CTRCD in allo-HSCT recipients.

**Methods:**

The records of 136 patients with haematological malignancies who underwent allo-HSCT at our institute were retrospectively reviewed. Early-onset CTRCD was defined as a decrease in left ventricular ejection fraction (LVEF) of ≥10% and an LVEF of ≤53% within 100 days after HSCT.

**Results:**

Early-onset CTRCD was diagnosed in 23 out of 136 included patients (17%), and the median duration from HSCT to CTRCD diagnosis was 24 (9–35) days. Patients were followed up for 347 (132–1268) days. In multivariate logistic regression analysis, cumulative doxorubicin dosage (each 10 mg/m^2^) and severity of acute graft-versus-host disease (GVHD/grade) were independent indicators of early-onset CTRCD (OR (95% CI) 1.04 (1.00 to 1.07); p=0.032; OR (95% CI) 1.87 (1.19 to 2.95), p=0.004, respectively). The overall and primary disease death rates were significantly higher in allo-HSCT recipients with early-onset CTRCD than in those without early-onset CTRCD (HR (95% CI) 1.98 (1.11 to 3.52), p=0.016; HR (95% CI) 2.96 (1.40 to 6.29), p=0.005, respectively), independent of primary disease type, remission status and transplantation type.

**Conclusions:**

Severe acute GVHD and higher cumulative anthracycline are two significant determinants of early-onset CTRCD. Early-onset CTRCD following allo-HSCT regulates survival in patients with haematological malignancies.

**WHAT IS ALREADY KNOWN ON THIS TOPIC**

- Haematopoietic stem cell transplantation (HSCT) is a key treatment for refractory haematological malignancies.
- The preconditioning therapy and cell transplantation can cause various complications that make patient management difficult and worsen prognosis.
- Cancer therapy-related cardiac dysfunction (CTRCD) following HSCT can be a serious complication; however, the clinical implications of early-onset CTRCD in recipients of HSCT remain unclear.

**WHAT THIS STUDY ADDS**

- Severe acute graft-versus-host disease (GVHD) and higher cumulative anthracycline dose are independent risk factors for early-onset CTRCD after allogeneic HSCT.
- Early-onset CTRCD aggravates the prognosis of patients with haematological malignancies who have undergone allogeneic HSCT.

**HOW THIS STUDY MIGHT AFFECT RESEARCH, PRACTICE AND/OR POLICY**

- Adequate immunosuppression for GVHD and preconditioning regimen alternative for anthracycline might preserve cardiac function.
- Monitoring of cardiac function and early intervention or prophylactic therapy for heart failure have a potential to improve prognosis of the patients who undergo allogeneic HSCT.

## Introduction

Haematopoietic stem cell transplantation (HSCT) is an effective therapy for malignant and non-malignant haematopoietic disorders. Its outcomes have dramatically improved following advances in transplantation procedures and supportive care, resulting in more long-term survivors who have undergone HSCT.^1^ In HSCT survivors, the incidence of cardiovascular diseases—such as heart failure (HF) and coronary artery disease—as well as their risk factors, is higher than that in the general population.^2 3^ High cardiovascular mortality in HSCT recipients suggests the importance of early cardiotoxicity detection in these patients.^2^ In patients who have undergone autologous HSCT, cardiotoxicity is mainly attributed to the direct toxic effects of the conditioning regimen; in recipients who have undergone allogeneic HSCT (allo-HSCT), it is also caused by the indirect effects of graft-versus-host disease (GVHD) and immunosuppressive therapy.^4 5^ As a cause of cardiac dysfunction in HSCT recipients, anthracyclines have well-known dose-dependent cardiotoxicity^3^; however, there is scarcity of evidence regarding other causes of cardiac dysfunction following HSCT. Since cardiac dysfunction after HSCT can be potentially lethal, its early appropriate management is considered critical for patient survival. Therefore, the relevant clinical factors for and the impact of early-onset cardiac dysfunction on patient prognosis need to be investigated.^6 7^

Recently, left ventricular systolic dysfunction after cancer therapy, detected by a decrease in left ventricular ejection fraction (LVEF), was defined as cancer therapy-related cardiac dysfunction (CTRCD). Therefore, periodic monitoring of LVEF via echocardiography is recommended for patients with cancer.^8 9^ Although several reports have described cardiotoxic events after HSCT,^10 11^ a definition for CTRCD in a recent consensus was not employed, and the relationship between left ventricular dysfunction at an early period following allo-HSCT and mortality has not been elucidated in these studies. Thus, we sought to clarify the incidence of early-onset CTRCD and its predictors and their effect on patient prognosis.

## Methods

### Study design

**Figure 1:**
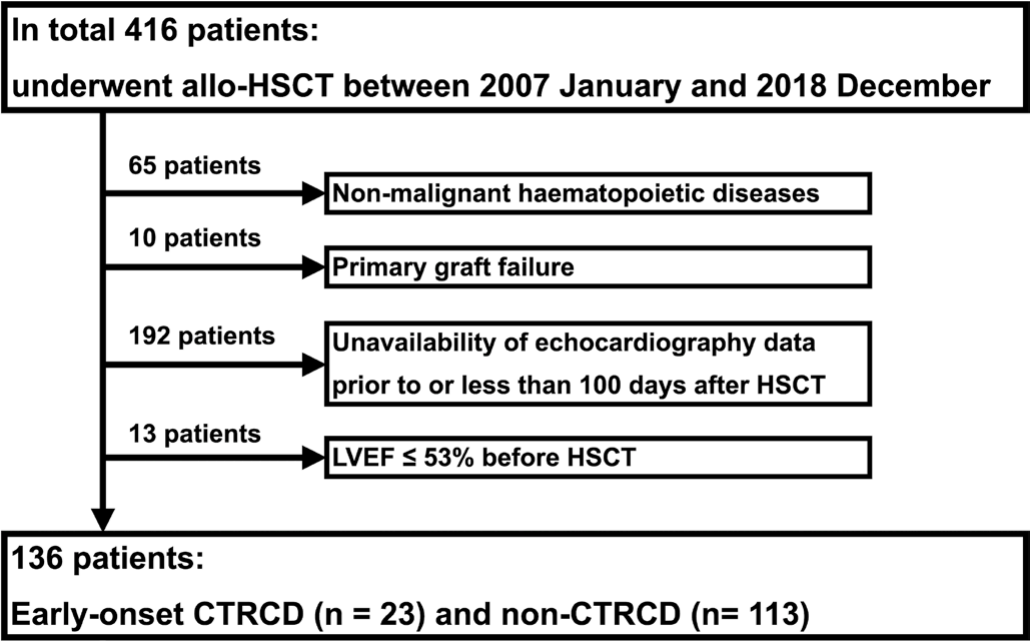
Patient screening and enrolment. The cohort comprised 416 consecutive patients who underwent allo-HSCT. patients who met at least one of the four exclusion criteria were excluded. A total of 136 patients (early-onset CTRCD, n=23; non-CTRCD, n=113) were included in the analysis. allo-HSCT, allogeneic haematopoietic stem cell transplantation; CTRCD, cancer therapy-related cardiac dysfunction; HSCT, haematopoietic stem cell transplantation; LVEF, left ventricular ejection fraction.

This single-centre, retrospective study included 416 consecutive patients who underwent allo-HSCT for haematological disease between January 2007 and December 2018 at Kyushu University Hospital. Patients with benign haematopoietic disease (n=65) in whom neutrophils were not engrafted after allo-HSCT (n=10), those whose echocardiograms had not been recorded prior to HSCT or within 100 days after HSCT (n=192), and those whose LVEF was ≤53% at the time of HSCT (n=13) were excluded; therefore, 136 patients were eligible for inclusion in the analysis (figure 1). The median follow-up period was 347 (132 to 1268) days.

Since the aim of this study was to investigate the predictors of early-onset CTRCD and its impact on patient prognosis, candidates for the predictors of early-onset CTRCD included common cardiac risk factors, anthracycline dosage and haematologically important factors for the management of patients who underwent HSCT. Overall survival (OS), treatment-related mortality and death due to underlying diseases were evaluated as prognostic indicators. The protocol of this study was approved by the internal review board (approval number: 2019–339), and patient consent was obtained via an opt-out method. This was a retrospective observational study, and patients or the public could not be involved in the design, conduct, reporting or dissemination plans of our study. All procedures performed in this study were in accordance with the principles of the updated Declaration of Helsinki (2013).

### Definitions

Growing evidence suggests that cardiooncology is a contemporary, diverse and challenging field of clinical science, and terminology relating to this field is somewhat confusing; therefore, we defined the terminology used in this study.

Early-onset CTRCD was defined as CTRCD occurring within 100 days after allo-HSCT; CTRCD was defined as a decrease in LVEF by ≥10% from baseline LVEF and an LVEF of ≤53% after HSCT, irrespective of baseline LVEF.^9^ The Teichholz method was adopted as the method to calculate LVEF. Echocardiography was performed and assessed by experienced cardiologists and echocardiographers. Baseline LVEF was assessed before the first chemotherapy session or at the first echocardiography at our hospital. Symptoms of HF were defined as dyspnoea or body oedema without other causes that can sufficiently explain the symptoms.

Cardiac risk factors include diabetes, hypertension, dyslipidaemia and current smoking.^12^ Creatinine clearance at HSCT was calculated using the Cockcroft-Gault equation. Cumulative anthracycline doses were converted to doxorubicin equivalents using conversion factors of 0.83, 0.67, 5.0 and 4.0 for daunorubicin, epirubicin, idarubicin and mitoxantrone, respectively.^13^ The myeloablative conditioning regimen was defined as a conditioning regimen with total body irradiation at ≥5 Gy in a single dose, ≥8 Gy in fractionated doses or an oral busulfan equivalent of ≥8 mg/kg with cyclophosphamide or melphalan.^14 15^

The disease status of haematological malignancies evaluated at the time of HSCT was categorised as complete remission (CR) or non-CR, irrespective of the status following prior HSCT. Disease relapse was defined as a haematologically detected relapse. Acute GVHD was defined as GVHD occurring within 100 days after HSCT and graded according to the standard criteria.^16^ Tacrolimus and cyclosporine were mainly administered for GVHD prophylaxis; other agents for GVHD prophylaxis included prednisolone, mycophenolate mofetil and methotrexate. Since tacrolimus can cause cardiomyopathy, it was incorporated as a variable. Treatment-related death was defined as death by GVHD, infection or other cancer therapy-related conditions, such as thrombotic microangiopathy and secondary cancer.

### Statistics analysis

Continuous variables are presented as medians (IQR) and compared between groups using the Mann-Whitney U test. Discrete variables were analysed with a contingency table using Fisher’s exact test. Patient characteristics were analysed using logistic regression analysis. Additionally, multiple logistic regression analyses were performed to elucidate the independent values. OS was summarised using the Kaplan-Meier method with the log-rank test, and Cox proportional hazards models, adjusted for several independent clinical factors, were used to estimate mortality. Independent factors with a p value of <0.05 in the univariate analysis, or those considered clinically relevant, were included in the multivariate analysis; factors with a high correlation (>0.6) were excluded as covariate factors to avoid multicollinearity. All multivariate analyses were conducted after confirming the fitting of models. Statistical significance (p) was set at <0.05. All statistical calculations were performed using JMP PRO V.15.1.0 (SAS Institute, Cary, North Carolina).

## Results

### Patient characteristics

Early-onset CTRCD was detected in 23 of the 136 patients (17%); symptomatic HF was observed in 11 of them, including three severe HF required ventilatory support. The median duration from HSCT to CTRCD diagnosis was 24 (9 to 35) days.

The median age of the included patients was 51 (37 to 61) years at the time of HSCT, and there was no difference in age between the CTRCD and non-CTRCD groups (table 1). Creatinine clearance was significantly lower in the CTRCD group than in the non-CTRCD group (p=0.026). Baseline LVEF and other echocardiographic parameters were comparable in the two groups (online supplemental table 1). Proportion of the patients with renin-angiotensin system inhibitors or beta-blockers before HSCT was also similar between the two groups (p=0.311). There was no difference in cardiac biomarkers prior to HSCT.

10.1136/openhrt-2022-002007.supp1 Supplementary data

**Table 1:** Patient characteristics

	Total (n=136)	CTRCD (n=23)	Non-CTRCD (n=113)	P value*
Age, years	51 (37–61)†	52 (36–61)	51 (37–61)	0.965
Male	75 (55)	9 (39)	66 (58)	0.110
CCr, mL/min	100 (81–123)	89 (63–101)	103 (82–127)	0.026
Cardiac risk factors				
Diabetes	16 (12)	4 (17)	12 (11)	0.475
Hypertension	18 (13)	4 (17)	14 (12)	0.507
Dyslipidaemia	11 (8)	1 (4)	10 (9)	0.690
Current smoker	34 (24)	4 (17)	3 (26)	0.437
Cardiac risk factors≥2	34 (25)	4 (17)	30 (27)	0.283
DXR before HSCT				
Cumulative DXR dose, mg/m^2^	231 (175–359)	296 (180–400)	208 (150–344)	0.087
Cumulative DXR dose ≥250 mg/m^2^	60 (44)	13 (57)	47 (42)	0.250
Baseline LVEF, %	68 (63–73)	66 (64–70)	69 (63–74)	0.158
RAS inhibitors or beta-blockers	2 (1)	1 (4)	1 (1)	0.311
Cardiac biomarker‡				
Brain natriuretic peptide, pg/ml	41 (12–73)	23 (11–157)	41 (13–67)	0.908
Troponin T, 10^-2^ ng/mL	1.0 (0.4–2.5)	2.3 (0.5–5.3)	1.0 (0.4–1.5)	0.428
Primary disease				
Leukaemia	98 (72)	16 (70)	82 (73)	0.801
Acute myeloid leukaemia	60 (44)	13 (57)	47 (42)	0.250
Acute lymphoblastic leukaemia	21 (15)	3 (13)	18 (16)	1.000
Chronic myeloid leukaemia	2 (1)	0 (0)	2 (2)	1.000
Other type leukaemia	15 (11)	0 (0)	15 (13)	0.074
Lymphoma	35 (26)	6 (26)	29 (26)	1.000
Plasma cell neoplasm	3 (2)	1 (4)	2 (2)	0.429
History of prior HSCT	34 (27)	9 (39)	28 (25)	0.199
Non-CR status at HSCT	89 (65)	18 (78)	71 (63)	0.229
Source of HSCT				
Bone marrow	58 (43)	8 (35)	50 (44)	0.491
Cord blood	34 (25)	7 (30)	27 (24)	0.598
PBSC	44 (32)	8 (35)	36 (32)	0.801
Haploidentical donor	13 (10)	3 (13)	10 (9)	0.461
HLA mismatch ≥one locus	81 (60)	17 (74)	64 (57)	0.163
Conditioning regimen with MAC	51 (38)	5 (22)	46 (41)	0.102
HCT-CI ≥3 points	15 (11)	5 (22)	10 (9)	0.135
GVHD prophylaxis including tacrolimus	107 (79)	16 (70)	91 (81)	0.268
Acute GVHD ≥Grade III	24 (18)	8 (35)	16 (14)	0.032

*CTRCD versus non-CTRCD.

†Values are presented as n (%) or median (25th to 75th percentile).

‡Values before HSCT, 60 in brain natriuretic peptide and 113 in troponin T were missing values.

CCr, creatinine clearance; CR, complete remission; CTRCD, cancer therapy-related cardiac dysfunction; DXR, doxorubicin; GVHD, graft-versus-host disease; HCT-CI, haematopoietic cell transplantation-specific comorbidity index; HLA, human leukocyteleucocyte antigen; HSCT, haematopoietic stem cell transplantation; LVEF, left ventricular ejection fraction; MAC, myeloablative conditioning; PBSC, peripheral blood stem cell; RAS, renin-angiotensin system.

Underlying diseases included acute myeloid (n=60), acute lymphoblastic leukaemia (n=21), chronic myeloid (n=2) and other types of leukaemia (n=15) as well as malignant lymphoma (n=35) and plasma cell neoplasm (n=3). Eighty-nine patients (65%) were categorised as non-CR at the time of HSCT. The sources of transplanted cells were bone marrow (n=58), cord blood (n=34) and peripheral blood (n=44) stem cells, including those from haploidentical donors (n=13). All recipients who underwent haploidentical peripheral blood stem cell transplantation had a non-CR status. The incidence of high-grade acute GVHD (grade III or IV) was significantly higher in the CTRCD group than in the non-CTRCD group (35% vs 14%, p=0.032).

### Change in LVEF after transplantation

In all participants, LVEF decreased from 68% (63%–73%) to 63% (56%–72%) after HSCT (p<0.001). LVEF after HSCT was 41% (26%–50%) in patients with CTRCD and 66% (60%–73%) in those without CTRCD (p<0.0001) (online supplemental figure 1). LVEF reduction (LVEF at baseline—LVEF after HSCT) was 26% (17%–40%) in patients with CTRCD and 3% (-2%–8%) in those without CTRCD (p<0.001).

### Clinical indicators of early-onset CTRCD

Among clinical factors, the haematopoietic cell transplantation-specific comorbidity index (HCT-CI; /point) was found to be significant predictors of early-onset CTRCD in univariate analysis (OR, 1.56; 95% CI 1.02 to 2.38; p=0.042) (table 2). Remarkably, the incidence of early-onset CTRCD was significantly associated with the severity of acute GVHD (grade; OR, 4.42; 95% CI 1.71 to 11.40; p=0.002). In addition, LVEF reduction was greater in patients with high-grade acute GVHD than in those with low-grade (grade I or II) GVHD (7^2–24^ vs 3 (−2–10], p=0.045) (online supplemental figure 2). In multivariate analysis, cumulative doxorubicin dose (each 10 mg/m^2^; OR, 1.04; 95% CI 1.00 to 1.07; p=0.032) and the grade of acute GVHD (grade; OR, 1.87; 95% CI 1.19 to 2.95; p=0.004) had a significant association with early-onset CTRCD (table 2). This result was robust in multiple logistic regression models adjusting for age and sex (online supplemental table 2).

**Table 2:** Univariate and age-adjusted multivariate analysis of risk factors for early-onset CTRCD after HSCT

	Univariate analysis		Multivariate analysis	
	OR (95% CI)	P value	OR (95% CI)	P value
Age	1.00 (0.97 to 1.04)	0.871		
Male	0.46 (0.18 to 1.15)	0.095		
CCr, /10 mL/min	0.86 (0.75 to 1.00)	0.052	0.88 (0.76 to 1.03)	0.098
Cardiac risk factors≥2	1.95 (0.56 to 6.78)	0.292		
Cumulative DXR dose, /10 mg/m^2^	1.03 (1.00 to 1.07)	0.058	1.04 (1.00 to 1.07)	0.032
RAS inhibitors or beta-blockers	5.09 (0.31 to 84.50)	0.256		
Leukaemia	0.86 (0.32 to 2.30)	0.770		
History of prior HSCT	1.95 (0.76 to 5.00)	0.163		
Non-CR status at HSCT	2.13 (0.74 to 6.16)	0.163		
Source of HSCT		0.783		
Bone marrow (reference)	1.00	–		
Cord blood	1.62 (0.53 to 4.95)	–		
PBSC	1.39 (0.48 to 4.05)	–		
Haploidentical PBSCT	1.55 (0.39 to 6.12)	0.536		
HLA mismatch ≥one locus	2.17 (0.80 to 5.91)	0.130		
Conditioning regimen with MAC	0.40 (0.14 to 1.17)	0.094		
HCT-CI, /point	1.56 (1.02 to 2.38)	0.042	1.38 (0.86 to 2.19)	0.177
GVHD prophylaxis including tacrolimus	0.51 (0.20 to 1.29)	0.155		
Acute GVHD, /grade	4.42 (1.71 to 11.40)	0.002	1.87 (1.19 to 2.95)	0.004

CCr, creatinine clearance; CR, complete remission; CTRCD, cancer therapy-related cardiac dysfunction; DXR, doxorubicin; GVHD, graft-versus-host disease; HCT-CI, haematopoietic cell transplantation-specific comorbidity index; HLA, human leucocyte antigen; HSCT, haematopoietic stem cell transplantation; LVEF, left ventricular ejection fraction; MAC, myeloablative conditioning; PBSC, peripheral blood stem cell; PBSCT, peripheral blood stem cell transplantation; RAS, renin-angiotensin system.

### Impact of early-onset CTRCD on prognosis

**Figure 2:**
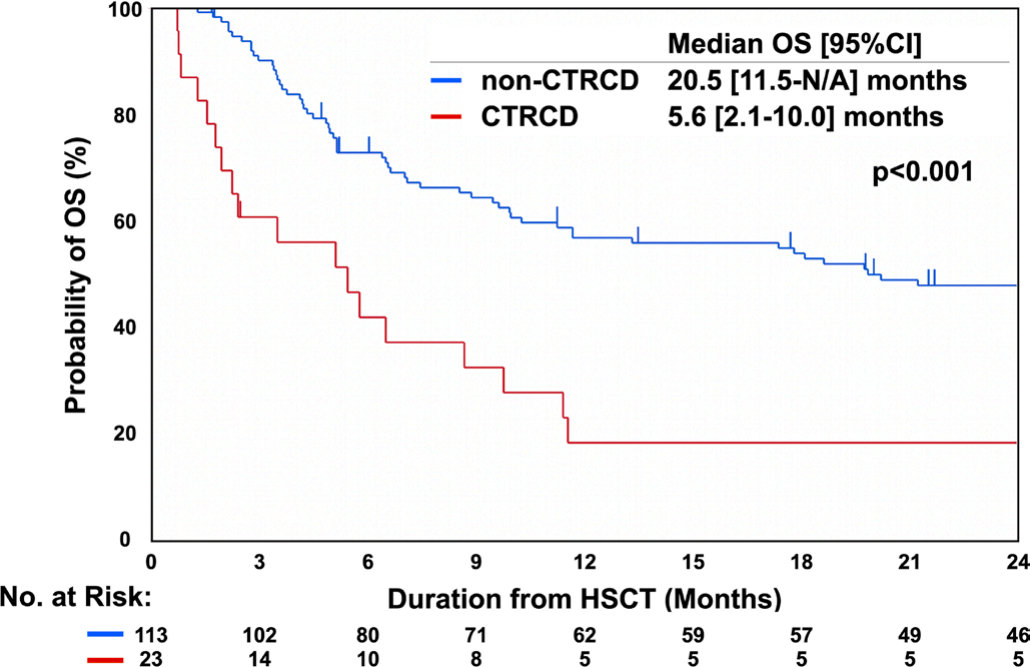
OS with respect to CTRCD. The median OS was significantly shorter in the CTRCD group than in the non-CTRCD group (p<0.001). CTRCD, cancer therapy-related cardiac dysfunction; HSCT, haematopoietic stem cell transplantation; N/A, not available; OS, overall survival.

The OS rate was significantly lower in recipients with early-onset CTRCD (median OS, 5.6 months; 95% CI 2.1 to 10.0 months) than in those without early-onset CTRCD (median OS: 20.5 months, 95% CI 11.5 to (not available) months; p<0.001; figure 2); additionally, early-onset CTRCD exaggerated overall death in unadjusted analysis (HR: 3.30, 95% CI 2.01 to 5.41; p<0.001; table 3). Among other underlying factors, a lower creatinine clearance level (p=0.012), non-leukaemia as the primary disease (p=0.038), non-CR status at HSCT (p=0.001), haploidentical peripheral blood stem cell transplantation (p=0.004), HCT-CI (/point; p<0.001) and the grade of acute GVHD (grade; p=0.006) were also found to significantly increase the risk of mortality (online supplemental table 3). Early-onset CTRCD significantly increased the risk of overall death even after adjusting for baseline backgrounds including age, sex, creatinine clearance, primary disease type, CR status, source of HSCT, HCT-CI and acute GVHD (HR, 1.98; 95% CI 1.11 to 3.52; p=0.016; table 3). In the CTRCD group, patients with HF symptoms had a poorer survival rate than asymptomatic patients (median OS, 2.4 (0.9 to 8.9) months vs 8.8 (1.9 to 26.2) months; p=0.006; online supplemental figure 3). HF medications (beta-blockers, angiotensin-converting enzyme inhibitors or angiotensin II receptor blockers) were initiated in 14 patients (61%) in the CTRCD group until the end of follow-up, and no significant difference in OS was observed between patients with CTRCD with HF medications and those without HF medications (median OS, 5.8 (1.4 to 11.6) months vs 2.5 (0.8 to 27.8) months; p=0.943).

**Table 3:** Association of CTRCD with overall or cause-specific death

	Unadjusted analysis		Adjusted analysis	
	HR (95% CI)	P value	HR (95% CI)	P value
Overall death	3.30 (2.01 to 5.41)	<0.001	1.98 (1.11 to 3.52)*	0.016
Primary disease death	3.91 (1.97 to 7.77)	<0.001	2.96 (1.40 to 6.29)†	0.005
Treatment-related death	2.77 (1.35 to 5.69)	0.005	1.78 (0.84 to 3.78)‡	0.131

*Adjusted for age, sex, CCr, primary disease type (leukaemia or not), CR status, source of HSCT (haploidentical PBSCT or not), HCT-CI (/point) and acute GVHD (/grade).

†Adjusted for age, sex, CCr, primary disease type (leukaemia or not), CR status, source of HSCT (haploidentical PBSCT or not), HCT-CI (/point).

‡Adjusted for age, sex, HCT-CI (/point) and acute GVHD (/grade).

CCr, creatinine clearance; CR, complete remission; CTRCD, cancer therapy-related cardiac dysfunction; GVHD, graft-versus-host disease; HCT-CI, haematopoietic cell transplantation-specific comorbidity index; PBSCT, peripheral blood stem cell transplantation.

Regarding the cause of death, primary disease and treatment-related deaths were observed in 39 (29%) and 42 (31%) patients, respectively (online supplemental table 4). Early-onset CTRCD significantly increased the risk of primary disease death in both unadjusted (HR, 3.91; 95% CI 1.97 to 7.77; p<0.001) and adjusted analysis (HR, 2.96; 95% CI 1.40 to 6.29; p=0.005) (table 3, online supplemental table 5). As the number of events was relatively small, association of early-onset CTRCD and the risk of primary disease death was confirmed by multiple adjustment models (online supplemental table 6). The primary disease in 14 patients (61%) in the CTRCD group and 41 patients (36%) in the non-CTRCD group relapsed after HSCT in this study. The proportion of patients who relapsed after HSCT and underwent retransplantation was significantly lower in the CTRCD group than in the non-CTRCD group (0% vs 23%, p=0.046). Early-onset CTRCD increased the risk of treatment-related death (HR, 2.77; 95% CI 1.35 to 5.69; p=0.005) in unadjusted analysis; however, the association was not significant after adjustment for age, sex, HCT-CI and acute GVHD (HR, 1.78; 95% CI 0.84 to 3.78; p=0.131) (table 3, (online supplemental table 7).

## Discussion

This study elucidated the predictors and prognostic impact of early-onset CTRCD after allo-HSCT in patients with haematological malignancies, and two major findings were identified. First, severe acute GVHD, in addition to high-dose cumulative anthracycline, was a significant indicator of early-onset CTRCD. Second, patients with early-onset CTRCD had insufficient OS with a higher primary disease mortality than those without early-onset CTRCD.

### Early-onset CRTCD and its indicators

Hertenstein *et al*^17^ reported that LVEF reduced less than 55% in 13% of patients who underwent HSCT within 3 months after transplantation, which is a comparable rate to the incidence of early-onset CTRCD in this study. Another study showed that 30% and 6% of patients who underwent HSCT developed clinically diagnosed HF and severe acute cardiomyopathy, respectively.^10^ These results showed that myocardial injury and subsequent left ventricular dysfunction are not rare and may be a limiting factor for HSCT.

Notably, grade of acute GVHD had a significant association with early-onset CTRCD. The graft versus host reaction was reported to have direct cardiotoxicity through donor T-cell infiltration into the myocardium as well as indirect toxicity due to cytokine release.^18^ We previously reported that serum tumour necrosis factor-α and interleukin-2 levels were elevated in acute GVHD.^19^ These cytokines were shown to aggravate cardiac function,^20^ whereas the immune response was suggested to be implicated in the pathogenesis of dilated cardiomyopathy.^21^ Additionally, the frequency of regulatory T cells and suppressive reaction of responder T cells decreased in patients with dilated cardiomyopathy.^22 23^ An inverse correlation between the number of regulatory T cells and the development of acute GVHD has also been reported in the previous studies.^24^ Therefore, impaired function of regulatory T cells in acute GVHD patients may lead to the activation of responder T cell-mediated immune reactions and subsequent inflammation in the myocardium, thereby inducing cardiac dysfunction. Since cases of acute GVHD-related cardiac dysfunction—recovered by increasing immunosuppressive therapy—have been reported,^18^ the optimisation of immunosuppressive drugs in patients with acute GVHD with CTRCD has the potential to improve patient prognosis by ameliorating cardiac function. Further research is needed to clarify the mechanism of GVHD in the development of CTRCD.

Our study also demonstrated an association between the cumulative dose of anthracycline and early-onset CTRCD. Several mechanisms of anthracycline-related cardiomyopathy have been suggested, such as oxidative stress, redox cycling by iron complexes and genetic variants.^25^ A cumulative anthracycline dose of ≥250 mg/m^2^ has been demonstrated to increase the risk of HF by up to 10-fold.^3 11^ This dose dependency of anthracycline-induced cardiotoxicity was also confirmed in our study, which targeted the early phase following HSCT. As an alternative to anthracycline in a preconditioning regimen, the use of molecular targeted therapy, which is generally less cardiotoxic than anthracycline, may reduce adverse cardiac events following HSCT and should be considered.

### Early-onset CTRCD as a prognostic factor

Early-onset CTRCD increased the risk of overall death by two-fold, and the HR for overall death was higher in patients with early-onset CTRCD than in those with a non-CR status. This suggests that early-onset cardiac dysfunction is a highly important determinant of recipient survival. As a cause of death, early-onset CTRCD has been shown to increase the risk of primary disease death more than double. One of the possible reasons for the high primary disease mortality in patients with CTRCD is the undertreatment of the primary disease. Treatment for refractory haematological malignancies after HSCT is performed with much difficulty due to decreased performance status, impaired immune system and tissue damage—including to the cardiovascular system—during prior treatment.^16^ As anthracycline should be reduced or discontinued in patients with cardiac dysfunction, patients with refractory haematological malignancies accompanied by early-onset CTRCD cannot receive sufficient treatment for underlying disease.^26^ In fact, none of the patients with early-onset CTRCD in our cohort underwent additional HSCT, even after relapse.

Early detection and treatment of cardiac dysfunction are of great significance to avoid undertreatment of haematological malignancies caused by HF. Repeated examinations of biomarkers such as troponin and echocardiographic parameters including global longitudinal strain may be useful for early detection of cardiac injury.^8 9^ In this study, the prognosis of patients with HF symptoms was extremely poor, and the difference in OS between CTRCD patients who received or did not receive HF medications was not statistically significant. These results suggest that initiation of HF treatment after the onset of symptomatic HF is too late to improve patient outcomes. Combined treatment with a renin-angiotensin system inhibitor and a beta-blocker is proved to preserve LVEF and reduce cardiac events in patients with haematopoietic malignancies by the OVERCOME (prevention of left ventricular dysfunction with enalapril and carvedilol in patients submitted to intensive chemotherapy for the treatment of malignant hemopathies) trial^27^; however, delayed initiation of HF treatment reduces therapeutic efficacy.^28^ Further research with a defined treatment protocol is needed to clarify the appropriate time to start the treatment of HF for patients with CTRCD.

A recent study suggested that HF is induced or aggravated via inflammation provoked by somatic mutations associated with clonal haematopoiesis of indeterminate potential (CHIP), such as tet methylcytosine dioxygenase 2 (TET2) and deoxyribonucleic acid methyltransferase 3 alpha (DNMT3A).^29^ Patients with a CHIP mutation had a low survival rate, possibly due to anthracycline resistance^30^; therefore, the possibility that CHIP acts as a common background for cardiac dysfunction after HSCT and the refractoriness of haematological malignancies should be investigated in future studies.

### Limitations

This study has some limitations. First, LVEF measured by the Teichholz method, which allows a potential off-axis measurement and has a relatively low accuracy of intra-LV volume presumption, was adopted. This is because LVEF from biplane image could not be measured in part of the recipients because of the difficulty in the apex approach in the lateral position due to poor physical status. Although CTRCD in this study was defined by only LVEF, detection of cardiac dysfunction by only LVEF poses a potential limitation as LVEF can be affected by volume status, preload and afterload. Subtle deterioration in LV systolic function may be better detected by the global longitudinal strain method.^31^ Second, this was a single-centre retrospective study with a limited number of patients, and patients without echocardiography before or after HSCT were excluded. As echocardiography was performed at a physician’s discretion, participant enrolment may have potentially led to a selection bias. The result that OS of the participants in this study was lower than that in the previous report of allogeneic HSCT^17^ might reflect higher proportion of patients with a serious condition in our participants. However, the incidences of CTRCD and severe acute GVHD were comparable to those in previous reports,^17 32 33^ suggesting that the participants in this study may not have been highly restricted distribution. Third, echocardiography was not performed in predefined schedule. To limit heterogeneity regarding the timing of echocardiography after HSCT, only cases who underwent echocardiography within 100 days after HSCT were included in this study. Forth, HF treatment was not performed using a predetermined protocol. Nevertheless, our study is one of the few studies limited to patients with malignant haematopoietic diseases who underwent allo-HSCT. Our finding that CTRCD defined by criteria reported in a recent consensus report determined the survival of HSCT recipients emphasised the importance of performing serial echocardiography in these patients. Future multicentre prospective trials will compensate for the limitations of this study and contribute to the investigation regarding the aetiology of and appropriate treatment for CTRCD, which could lead to improved prognosis in patients undergoing HSCT.

## Conclusion

Our study identified severe acute GVHD as well as higher cumulative anthracycline as indicators of early-onset CTRCD. Additionally, early-onset CTRCD was an independent factor for poor prognoses with a higher primary disease mortality in recipients following allo-HSCT. Further research is needed to clarify the mechanism underlying the relationship between CTRCD and GVHD, an optimised regimen of less cardiotoxic cancer chemotherapy for patients with CTRCD and the appropriate time to initiate HF treatment in recipients of allo-HSCT.

## Data Availability

Data are available upon reasonable request.
