# Supplementary material for: Early-onset cardiac dysfunction following allogeneic haematopoietic stem cell transplantation
Source: Open Heart. 2022 May 19;9(1):e002007. doi: 10.1136/openhrt-2022-002007 (PMC9125755; doi:10.1136/openhrt-2022-002007)

Supplemental material

Early-Onset Cardiac Dysfunction Following Allogeneic Haematopoietic Stem Cell Transplantation

Shohei Moriyama[a], Mitsuhiro Fukata[a], Michinari Hieda[a], Taku Yokoyama[a], Goichi Yoshimoto[a], Hitoshi Kusaba[a], Yasuhiro Nakashima[b], Toshihiro Miyamoto[c], Toru Maruyama[d], Koichi Akashi[a]

[a]Department of Haematology, Oncology and Cardiovascular Medicine, Kyushu University Hospital, Fukuoka, Japan; [b]Department of Medicine and Bioregulatory Science, Graduate School of Medical sciences, Kyushu University, Fukuoka, Japan; [c] Department of Hematology, Faculty of Medicine, Institute of Medical Pharmaceutical and Health Sciences, Kanazawa University, Ishikawa, Japan; [d]Campus Life Health Centre, Kyushu University, Fukuoka, Japan

Supplemental Table 1. Baseline echocardiographic parameters

	CTRCD (n=23)	Non-CTRCD (n=113)	P-value*
Dimension			
LVEF (%)	65.8 (64.4-70.0)	69.1 (63.3-74.0)	0.158
LV end-diastolic diameter (mm)	47.0 (43.0-51.0)	47.0 (44.0-51.0)	0.914
LV end-systolic diameter (mm)	31.0 (27.0-32.0)	29.0 (25.0-32.0)	0.249
Left atrial diameter (mm)	32.5 (27.8-36.0)	34.0 (29.5-38.0)	0.245
Transmitral flow			
Peak E wave velocity (cm/s)	73 (57.4-82.8)	71.1 (59.0-88.8)	0.967
Peak A wave velocity (cm/s)	65.8 (51.1-81.3)	66.2 (53.2-77.9)	0.776
E/A ratio	1.2 (0.9-1.5)	1.1 (0.9-1.4)	0.401
Deceleration time (ms)	183.5 (134.0-214.0)	190.0 (158.0-227.3)	0.412
Tissue doppler			
e’ velocity (cm/s)	8.8 (6.6-11.1)	8.0 (6.5-9.6)	0.508
a’ velocity (cm/s)	9.2 (7.8-11.3)	9.3 (7.9-10.5)	0.897
s’ velocity (cm/s)	8.2 (6.9-9.9)	8.5 (7.3-9.5)	0.870
E/e’ ratio	8.0 (6.3-11.7)	8.6 (7.4-10.9)	0.569

Values are median (25th to 75th percentile). *: CTRCD vs. Non-CTRCD.

CTRCD, cancer therapy-related cardiac dysfunction; EF, ejection fraction; LV, left ventricular.

Supplemental Table 2. Age- and sex-adjusted multivariate analyses of risk factors for early-onset CTRCD after HSCT

	Model 1		Model 2		Model 3		Model 4		Model 5	
	Odds ratio [95% CI]	P	Odds ratio [95% CI]	P	Odds ratio [95% CI]	P	Odds ratio [95% CI]	P	Odds ratio [95% CI]	P
CCr, /10 ml/min	0.87 [0.74-1.03]	0.089	0.87 [0.73-1.02]	0.086	0.89 [0.74-1.03]	0.106	0.89 [0.71-0.99]	0.030	-	-
Cumulative DXR dose, /10 mg/m^2	1.03 [1.00-1.07]	0.082	-	-	-	-	-	-	1.03 [1.00-1.07]	0.045
Conditioning regimen with MAC	-	-	0.46 [0.15-1.43]	0.179	-	-	-	-	0.41 [0.13-1.28]	0.109
HCT-CI, /point	-	-	-	-	0.89 [0.87-2.10]	0.179	-	-	-	-
Acute GVHD, /grade	-	-	-	-	-	-	0.89 [1.19-2.92]	0.004	-	-

	Model 6		Model 7		Model 8		Model 9		Model 10	
	Odds ratio [95% CI]	P	Odds ratio [95% CI]	P	Odds ratio [95% CI]	P	Odds ratio [95% CI]	P	Odds ratio [95% CI]	P
CCr, /10 ml/min	-	-	-	-	-	-	-	-	-	-
Cumulative DXR dose, /10 mg/m^2	0.89 [1.00-1.07]	0.046	0.89 [1.01-1.08]	0.013	-	-	-	-	-	-
Conditioning regimen with MAC	-	-	-	-	0.46 [0.15-1.45]	0.172	0.43 [0.14-1.38]	0.141	-	-
HCT-CI, /point	0.89 [0.94-2.30]	0.095	-	-	0.89 [0.89-2.14]	0.147	-	-	0.89 [0.91-2.19]	0.125
Acute GVHD, /grade	-	-	0.89 [1.25-3.14]	0.002	-	-	0.89 [1.14-2.70]	0.008	0.89 [1.14-2.72]	0.008

CI, confidence interval; CR, complete remission; CTRCD, cancer therapy-related cardiac dysfunction; DXR, doxorubicin; GVHD, graft-versus-host disease; HCT-CI, haematopoietic cell transplantation-specific comorbidity index; HSCT, haematopoietic stem cell transplantation; LVEF, left ventricular ejection fraction; P, P value.

Supplemental Table 3. Unadjusted analysis for time from HSCT to overall death

	HR [95% CI]	P-value
Age	1.01 [0.99-1.03]	0.313
Male	0.95 [0.61-1.47]	0.815
CCr, /10 ml/min	0.92 [0.86-0.98]	0.018
Cardiac risk factors ≥ 2	0.71 [0.34-1.47]	0.354
Leukaemia	0.61 [0.38-0.97]	0.038
History of HSCT	1.53 [0.96-2.43]	0.072
Non-CR status at HSCT	2.33 [1.39-3.90]	0.001
Haploidentical PBSCT	2.65 [1.36-5.16]	0.004
HLA mismatch ≥ one locus	1.21 [0.77-1.90]	0.412
GVHD prophylaxis including tacrolimus	1.23 [0.70-2.15]	0.475
HCT-CI, /point	1.59 [1.28-1.97]	<0.001
Acute GVHD, /grade	1.37 [1.09-1.71]	0.006
Early-onset CTRCD	3.30 [2.01-5.41]	<0.001

Number of overall deaths: 81.

CCr, creatinine clearance; CI, confidence interval; CR, complete remission; CTRCD, cancer therapy-related cardiac dysfunction; GVHD, graft-versus-host disease; HCT-CI, haematopoietic cell transplantation-specific comorbidity index; HLA, human leukocyte antigen; HR, hazard ratio; HSCT, haematopoietic stem cell transplantation; PBSCT, peripheral blood stem cell transplantation.

Supplemental Table 4. Cause of death in patients with early-onset CTRCD

	All (n=136)	CTRCD (n=23)	Non-CTRCD (n=113)
All cause death, n (%)	81 (60)	22 (96)	59 (52)
Primary disease death, n (%)	39 (29)	12 (52)	27 (24)
Treatment-related death	42 (31)	10 (43)	32 (28)
GVHD, n (%)	7 (5)	3 (13)	4 (4)
Infection, n (%)	30 (22)	7 (30)	23 (2)
Others*, n (%)	5 (4)	0 (0)	5 (4)

CTRCD, cancer therapy-related cardiac dysfunction; GVHD, graft-versus-host disease.

*Others: other type cancer, thrombotic microangiopathy, sinusoidal obstruction syndrome/veno-occlusive disease, non-infectious pulmonary complications, cerebral and/or gastrointestinal haemorrhage.

Supplemental Table 5. Unadjusted analysis for time from HSCT to primary disease death

	HR [95% CI]	P-value
Age	1.00 [0.98-1.03]	0.767
Male	0.85 [0.45-1.59]	0.601
CCr, /10 ml/min	0.91 [0.82-1.00]	0.070
Cardiac risk factors ≥ 2	0.53 [0.16-1.73]	0.296
Leukaemia	0.48 [0.25-0.91]	0.026
History of HSCT	1.38 [0.70-2.73]	0.351
Non-CR status at HSCT	6.26 [2.22-17.68]	<0.001
Haploidentical PBSCT	4.32 [1.89-9.86]	<0.001
HLA mismatch ≥ one locus	1.62 [0.82-3.19]	0.167
GVHD prophylaxis including tacrolimus	0.82 [0.40-1.69]	0.600
HCT-CI, /point	1.59 [1.15-2.16]	0.004
Acute GVHD, /grade	0.93 [0.68-1.27]	0.635
Early-onset CTRCD	3.91 [1.97-7.77]	<0.001

Number of primary disease death: 39.

CCr, creatinine clearance; CI, confidence interval; CR, complete remission; CTRCD, cancer therapy-related cardiac dysfunction; GVHD, graft-versus-host disease; HCT-CI, haematopoietic cell transplantation-specific comorbidity index; HLA, human leukocyte antigen; HR, hazard ratio; HSCT, haematopoietic stem cell transplantation; PBSCT, peripheral blood stem cell transplantation.

Supplemental Table 6. Adjusted analyses of the association between early-onset CTRCD and time from HSCT to primary disease death

	HR [95% CI]	P-value
Model 1	3.50 [1.66-7.34]	0.001
Model 2	3.77 [1.85-7.70]	<0.001
Model 3	3.77 [1.84-7.71]	<0.001
Model 4	3.20 [1.50-6.82]	0.003
Model 5	3.09 [1.48-6.47]	0.003
Model 6	3.43 [1.60-7.36]	0.002
Model 7	2.84 [1.33-6.06]	0.007
Model 8	3.26 [1.60-6.64]	0.001

Model 1: adjusting age, sex, CCr, and disease type*.

Model 2: adjusting age, sex, disease status†, and source of HSCT‡.

Model 3: adjusting age, sex, source of HSCT‡, and HCT-CI.

Model 4: adjusting CCr, disease type*, disease status†, source of HSCT‡.

Model 5: adjusting CCr, disease type*, disease status†, and HCT-CI.

Model 6: adjusting CCr, disease type*, source of HSCT‡, and HCT-CI.

Model 7: adjusting CCr, disease status†, source of HSCT‡, and HCT-CI.

Model 8: adjusting disease type*, disease status†, source of HSCT‡, and HCT-CI.

*, leukaemia or not; † CR or not; ‡, haploidentical PBSCT or not.

CCr, creatinine clearance; CI, confidence interval; CR, complete remission; CTRCD, cancer therapy-related cardiac dysfunction; GVHD, graft-versus-host disease; HCT-CI, haematopoietic cell transplantation-specific comorbidity index; HR, hazard ratio; PBSCT, peripheral blood stem cell transplantation.

Supplemental Table 7. Unadjusted analysis for time from HSCT to treatment related death

	HR [95% CI]	P-value
Age	1.01 [0.99-1.04]	0.266
Male	1.06 [0.57-1.95]	0.857
CCr, /10 ml/min	0.93 [0.84-1.02]	0.122
Cardiac risk factors ≥ 2	0.88 [0.35-2.24]	0.788
Leukaemia	0.78 [0.40-1.53]	0.468
History of HSCT	1.67 [0.89-3.14]	0.111
Non-CR status at HSCT	1.28 [0.67-2.41]	0.453
Haploidentical PBSCT	1.38 [0.42-4.48]	0.597
HLA mismatch ≥ one locus	0.94 [0.51-1.73]	0.838
GVHD prophylaxis including tacrolimus	2.02 [0.80-5.15]	0.139
HCT-CI, /point	1.60 [1.17-2.14]	0.002
Acute GVHD, /grade	2.00 [1.45-2.79]	<0.001
Early-onset CTRCD	2.77 [1.35-5.69]	0.005

Number of primary disease death: 42.

CCr, creatinine clearance; CI, confidence interval; CR, complete remission; CTRCD, cancer therapy-related cardiac dysfunction; GVHD, graft-versus-host disease; HCT-CI, haematopoietic cell transplantation-specific comorbidity index; HLA, human leukocyte antigen; HR, hazard ratio; HSCT, haematopoietic stem cell transplantation; PBSCT, peripheral blood stem cell transplantation.

Supplemental Figure 1. LVEF change in CTRCD and non-CTRCD group

LVEF decreased from 66% (64%-70%) to 41% (26%-50%) in the early-onset CTRCD group ($p<0.001$) and from 69% (63%-74%) to 66% (60%-73%) in the non-early-onset CTRCD group ($p<0.001$) after HSCT (interaction-$p<0.001$).

CTRCD, cancer therapy-related cardiac dysfunction; HSCT, haematopoietic stem cell transplantation; LVEF, left ventricular ejection fraction

Supplemental Figure 2. Difference in LVEF reduction between patients with low-grade and high-grade acute GVHD

The reduction in LVEF was significantly higher in patients with high-grade acute GVHD than in patients with low-grade GVHD ($p=0.045$).

IQR, interquartile range; LVEF, left ventricular ejection fraction; GVHD, graft-versus-host disease

Supplemental Figure 3. OS with respect to the presence of heart failure symptoms in patients with CTRCD

In patients with CTRCD, the median OS was significantly shorter in patients with heart failure symptoms than in those without heart failure symptoms ($p=0.006$).

CI, confidence interval; CTRCD, cancer therapy-related cardiac dysfunction; HSCT, haematopoietic stem cell transplantation; OS, overall survival

Supplemental Figure 1

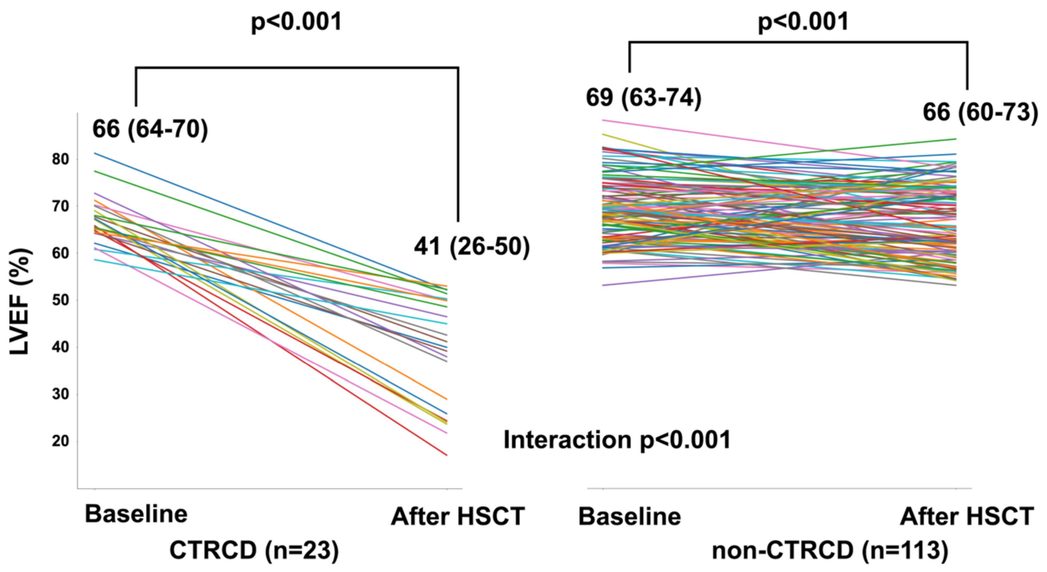

Supplemental Figure 2

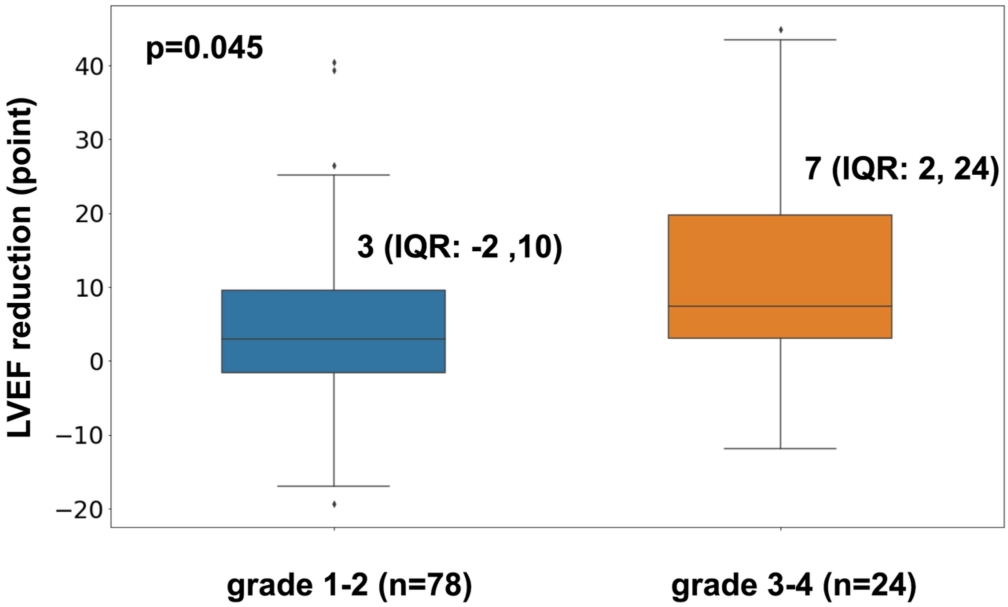

Supplemental Figure 3

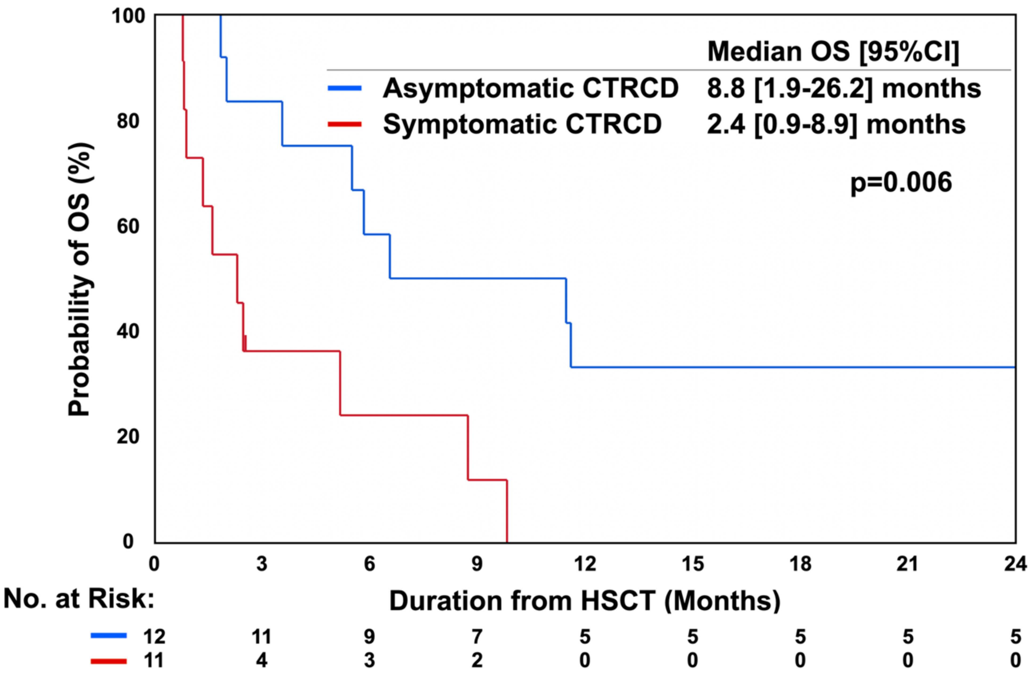

Supplement: Supplementary data [file openhrt-2022-002007supp001.pdf]
